# Prosocial Engagement and Health: Theoretical and Methodological Innovation

**DOI:** 10.1093/geroni/igaf122.202

**Published:** 2025-12-31

**Authors:** Seoyoun Kim, Cal Halvorsen, Nancy Morrow-Howell

## Abstract

All individuals should have opportunities for meaningful engagement, including volunteering and informal helping, across the life course. These prosocial activities foster purpose and connection, essential for well-being in later life. However, opportunities and benefits are shaped by structural factors such as socioeconomic status, race, and pre-existing health conditions. Research on volunteering’s health effects has often overlooked these complexities and lacked rigorous causal methodologies. To that end, this symposium presents cutting-edge research on the health benefits of prosocial engagement in later life. Kim examines the causal link between volunteering and cardiovascular biomarkers using g-computation with Health and Retirement Study (HRS) data (2005-2016), enhancing causal inference. Ryu investigates the relationship between volunteering and hypertension risk, stratified by insurance status, using the entropy balancing weights to mitigate selection bias and enhance estimate accuracy. Nemeth expands the discussion by analyzing formal and informal helping and their impact on functional recovery. Chen explores how helping others influences psychological well-being, employing ecological momentary assessment (EMA) to capture real-time emotional and behavioral responses. Han utilizes HRS data (1998-2020) to examine how changes in helping behaviors—both in role status and commitment—affect cognitive health over time, using an extended asymmetric fixed-effects model to address endogeneity and establish potential causal relationships. Morrow-Howell (discussant) will provide insights into the broader implications and future directions of this research. Together, these studies advance the field by integrating robust causal inference techniques, emphasizing inclusion, and leveraging innovative data collection methods to deepen our understanding of prosocial engagement and aging.

